# Temporal Polyethism and Worker Specialization in the Wasp, *Vespula germanica*

**DOI:** 10.1673/031.007.4301

**Published:** 2007-07-23

**Authors:** Christine R. Hurd, Robert L. Jeanne, Erik V. Nordheim

**Affiliations:** ^1^ Department of Zoology, University of Wisconsin-Madison; ^2^ Departments of Entomology and Zoology, University of Wisconsin-Madison; ^3^ Departments of Statistics and Forest Ecology and Management, University of Wisconsin-Madison

**Keywords:** social insects, age polyethism, division of labor, foraging, yellowjackets

## Abstract

Temporal polyethism is a common mechanism of worker specialization observed in social insect species with large colony sizes, *Vespula* wasp colonies consist of thousands of monomorphic workers, yet studies based on small cohorts of workers report that temporal polyethism is either weak or completely absent in different *Vespula* species. Concerned that the small sample size of these studies precluded detection of temporal polyethism, several hundred, known-age *Vespula germanica* (F.) (Hymenoptera: Vespidae) workers were studied. High variability was found in the sequence and diversity of tasks workers perform, suggesting that *V. germanica* colonies exhibit weak temporal polyethism. The most common order in which tasks were taken up was 1) nest work, 2) pulp foraging, 3) carbohydrate foraging, and 4) protein foraging. However, only 61% of the wasps performed more than two of the tasks during their lives. Thorax size had a significant negative effect on the age at first foraging, but the magnitude of the effect was small. The daily ratio of task generalists to specialists was relatively constant despite the high turnover of workers, growth of the colony, and the colony's transition from rearing worker larvae to rearing reproductives. Over the course of their lives, 43% of the workers averaged more than one kind of task performed per day. Life history traits are identified that may explain why vespines with large colonies use a generalist strategy of labor division rather than the specialist strategy observed in honey bees (*Apis mellifera*) and large colonies of wasps (*Polybia occidentalis*).

## Introduction

Worker specialization can improve the efficiency of task performance in social insects (Heinrich 1976; Oster and Wilson 1978; Seeley 1985; Jeanne 1986a; Cartar 1992; O'Donnell and Jeanne 1992). A common mechanism by which monomorphic workers differentiate into task specialists is temporal, or age, polyethism, in which workers tend to specialize sequentially on tasks over the course of their lives, rather than perform all tasks concurrently. The typical progression is from nest tasks to foraging tasks (Lindauer 1961; Wilson 1971; Seeley 1982, 1995; Jeanne 1986b, 1991). In addition, in age polyethic species the tendency of larger workers to forage at a younger age than smaller workers has been observed in ants (Wilson 1971; Mirenda and Vinson 1981), *Bombus* bees (Brian 1952) and the wasps *Polistes jadwigae* (Tsuchida and Itô 1991) and *Polybia occidentalis* (O'Donnell and Jeanne 1995). The size of *Vespula* workers increases as colony size increases, and larger workers may be more likely to forage than smaller workers (Spradbery 1965, 1973; Archer 1972); however, worker size has not been linked to earlier onset of foraging tasks.

The degree of temporal polyethism varies widely across social insect species and appears related to colony size (Wilson 1971; Jeanne 1999; Tomas and Elgar 2003). Colonies of ants, bees, wasps, and termites, consisting of thousands to millions of workers, divide colony labor among groups of highly-specialized workers (Möglich and Hölldobler 1974; Jeanne 1986a, 2003; Hölldobler and Wilson 1990; Seeley 1995; Crosland et al. 1997; Pie 2002). In contrast, workers in small colonies consisting of fewer than one hundred adults tend to be more generalized and to perform multiple kinds of tasks daily [seen in *Bombus* bees (Cameron and Robinson 1990), *Polistes* wasps (Post et al. 1988; Reeve 1991), and small colonies of the ant *Rhytidoponera metallica* (Thomas and Elgar 2003)].

One reason for this dichotomy may be that specialization leads to efficiency gains only in large colonies. For example, colonies of the wasp, *Polybia occidentalis*, typically consist of several hundred highly specialized workers. Small colonies of less than 50 workers suffered long delays in nest material transfer when specialist foragers had difficulty locating specialist receivers (Jeanne 1986a). All nest repair ceased when the delays grew exceptionally long. In contrast, colonies utilizing large numbers of material donors and receivers experienced shorter transfer times and reared more brood per capita.

*Vespula* species, such as *V. atropilosa*, *V. flaviceps*, *V. pensylvanica*, and *V. vulgaris* appear to be exceptions to the correlation between large colony size and worker specialization seen in other eusocial insects. Thousands of workers comprise these colonies, yet workers tend to be task generalists and only loosely follow a temporal polyethism schedule (Brian and Brian 1952; Potter 1964; Spradbery 1973; Akre et al. 1976; Matsuura and Yamane 1990). Montagner (1966) concluded that division of labor was virtually independent of age in *V. germanica*. This apparent lack of task specialization is curious, given the theoretical and empirical studies linking efficiency gains with task specialization in large colonies (Oster and Wilson 1978; Jeanne 1986a, 1986b; Anderson and Ratnieks 1999).

It is alternatively possible that studies reporting reduced specialization in *Vespula* species had an insufficient sample size to detect temporal polyethism. These studies were based on single-aged cohorts, generally consisting of fewer than 30 workers, in small, though not necessarily young, colonies (Brian and Brian 1952; Potter 1964; Akre et al. 1976). Montagner's study, for example, was based on only 20 workers (Montagner 1966). When a single cohort of identically-aged workers was observed over numerous days, an observed similarity in worker task switching may have been due to a change in the colony's needs rather than to a change in worker age.

The purpose of this study is to determine if temporal polyethism and worker specialization can be detected in large samples of consecutively-introduced, known-age workers in captive colonies of *V. germanica* (F.) (Hymenoptera: Vespidae). With continual observation of two colonies for up to nine hours per day for 37 days, the most detailed analysis of *Vespula* division of labor was obtained to date.

## Methods

A *V. germanica* colony was excavated in Madison, Wisconsin, in late July of 1998 and another in late July of 1999. Each colony was housed in a 43.0 cm × 42.5 cm × 4.1 cm observation box with a glass bottom and top that were kept covered when observations were not being made. The colony was confined to an outdoor screen tent (7 m × 3 m × 2.5 m) in the University of Wisconsin-Madison Arboretum. A large sheet of construction paper was provided as a source of nesting material. This paper was the colonies' only pulp source, and on it all pulp foragers could be easily observed. The colonies were constrained to forage on food provided inside the tent. Every morning at 0800 hours a plastic dinner plate containing a 1:2 honey-to-water solution, and a plate containing a mixture of cooked meats (ham, turkey, beef, or fish) were placed on a table inside the tent approximately 3 m from the nest. The honey-water and meats were continuously available until their removal at approximately 2100 hours each night. In addition, flies, crickets, and sweep-netted arthropods were spread on the table once a day. The live prey items were kept on the table by disabling them. With this experimental design, we attempted to minimize the variation in worker foraging rates that was due to differences in food source distance, quality, handling time, and search time. The goal was to discover the range of variability in workers' predispositions to perform certain kinds of tasks without those predispositions being influenced by fluctuations in the external environment.

Additional field assistants in 1999 were used to estimate the colony's size. The comb cells and top of the nest were photographed each morning before making the food available. At this time foragers were usually inactive, and so most of the workers were still inside the nest. The number of wasps seen in each image were counted.

### Establishing a set of known-age workers

To collect lifetime data on known-age individuals, one of the combs from the 1998 colony was placed in an indoor nestbox and incubated at ambient temperature. As workers emerged, they were marked on the thorax for individual recognition with Deco Color© paint pens and placed with their sisters and queen in the Arboretum colony on the night of their eclosion. Because the 1998 colony was small, the number of known-age individuals was increased by adding workers that emerged from incubated combs of three unrelated *V. germanica* colonies. Previous work on *Polybia occidentalis* wasps showed that workers less than 24 hours old are accepted into an unrelated colony and behave normally (Jeanne et al. 1988). Between 24 July and 3 September 1998, 1,194 marked workers were added: 84 were from the original colony (colony 1), and thus were sisters of the workers in the observation box; 171 were added from colony 2; 302 from colony 3, and 637 from colony 4.

The 1999 nest was larger. Two of its seven combs were incubated indoors. To the observation colony, a total of 607 known-age sisters that emerged from these combs between 23 July and 20 August were added. No unrelated workers were introduced. All analyses of age-based task performance are based solely on known-age workers.

### Carbohydrate-foraging data

To identify the colonies' carbohydrate foragers and measure their rates of foraging, the identity of every worker feeding on the honey solution and her time of feeding was recorded to the nearest minute. When an unmarked worker landed on the dish, she was captured, chilled, individually marked, and released minutes to hours later, at the end of the observation period. A worker's foraging rate was calculated as her (number of trips - 1) / (minutes between her first and last foraging trip) for each observation period. The number of trips - 1 is the number of times a forager delivered carbohydrate to the nest. In 1998 (28 Jul – 31 Aug) 1–2 hours of carbohydrate-foraging data were collected daily, alternating between mornings (1000 hrs) and afternoons (1500 hrs). In 1999 (27 Jul – 1 Sep) data were collected from 0800 hrs -1200 hrs, and for 15 of these 37 days (30 Jul – 13 Aug) data were collected throughout the afternoon (1200 hrs – 1700 hrs).

### Pulp-foraging and protein-foraging data

In both years, for 1–3 hours per day, and most often simultaneously with the carbohydrate-foraging data collection, an observer recorded the identity of all marked workers retrieving pulp (from a construction paper stand) and protein (cooked meats and sweep-netted arthropods spread on a table).

### Nest-work data

To identify workers that performed in-nest tasks, the activities of all marked workers found on the comb faces and backs were documented during four 15-minute scan samples taken daily in 1998, and three to nine 20-minute scan samples made daily in 1999. Workers that cleaned cells, cleared debris, fed workers, fed larvae, inspected cells, built cells or fanned the nest during these observation periods were recorded as performing nest work. Marked individuals in the nest not doing these tasks were recorded simply as being seen.

### Body-size data

During the last two weeks of the study seasons, marked foragers were randomly caught outside the nest and measured for body size. Live workers were placed in vacuum-sealed plastic bags, frozen at -50° C., oven-dried at 60° C for seven days, then weighed on a Cahn 29 automatic electrobalance to the nearest 0.1µg. The dorsal side of each worker's head and thorax were photographed using a Polaroid PDMC-2 video camera mounted on a Leica M275 stereo microscope at 25× magnification. Using Image Pro Plus image analysis software, head width (distance between the medial edges of the compound eyes at the posterior edge of the median ocellus), and thorax width (maximum width of the mesoscutum) were measured. 68 workers were measured in 1998 and 137 workers in 1999.

### Data analyses

Although workers' deaths were not recorded, worker lifespan was estimated for the 1999 colony in two ways. One estimate was based on all known-age workers that were seen either in the nest or foraging on at least three separate days and not seen after 24 August, one week before the study ended. A second estimate was based on known-age workers seen at least once and not seen after 24 August.

To determine if workers commenced the tasks of nest work, pulp foraging, protein foraging, and carbohydrate foraging in a serial fashion as they aged, rather than concurrently, the tasks were analyzed in pairs using only known-age wasps that had done both tasks. For each set of two tasks, workers were classified according to which of the tasks they had performed first. The binomial test (excluding ties) was used to test the null hypothesis that the proportion of workers in each group was 0.50, and thus workers had an equal probability of performing either task first (SAS Proc Freq) (SAS Institute Inc. 1999).

In a second approach, a subset of known-age workers that foraged for each material, pulp, protein, and carbohydrate, were evaluated at least three times in their lives. For the 32 workers meeting this criterion, a scoring method was developed that discerned patterns of task performance order. For each forager, each kind of task she performed was ranked in the order of its occurrence over the course of her foraging career. If she did multiple tasks on the same day, those tasks were given an average of the ranks for that day. For example, if she foraged for pulp on day one (defined as the first day we observed her foraging) and carbohydrate foraged on day two, she received a rank of 1.0 for pulp and 2.0 for carbohydrate. If on day four she foraged for carbohydrate and protein, she would receive a rank of 3.5 for carbohydrate and 3.5 for protein (the average of 3.0 and 4.0). Then the average rank for each material was calculated. The tasks performed most often later in a foraging career ended up with the highest ranks. In this example, the worker would receive an average rank of 1.0 for pulp, 2.75 (=(2.0 + 3-5)/2) for carbohydrate and 3.5 for protein. A value of one was arbitrarily chosen as the criterion of difference. If the tasks' average ranks differed by less than one, the tasks were considered to have been performed simultaneously. Different criterion values (other than one) were also considered and the same results were found. Thus, in our example, pulp foraging occurred prior to carbohydrate and protein foraging, while carbohydrate and protein foraging were performed concurrently. The numbers of wasps that performed pulp, protein, and carbohydrate foraging were then compared in the different possible foraging sequences.

To test the effect of body size on the rate at which a worker progressed through the task sequence, the age of first foraging for each material was regressed separately on each measure of body size: weight, head size, thorax size, and the ratio of thorax size to weight. A multiple regression of age of first foraging for a material type was performed on all measures of body size combined.

It is possible that a worker's daily expenditure of foraging effort varies little over the course of her life. A worker could be expected to make more trips for a particular material on days when she specialized on collecting that material and fewer trips for that material on days she foraged for additional materials or worked in the nest. Alternatively, foraging specialization may have no effect on the number of trips a worker makes for a particular material. To test these hypotheses, the number of carbohydrate-foraging trips each worker made on the days she foraged only for carbohydrate was regressed with the number of carbohydrate-foraging trips made on days when she also worked in the nest or foraged for pulp or protein.

Worker task specialization was examined at the individual level and the colony level using the 1999 data. The frequency distributions of the number of workers observed doing 0, 1, 2, 3, and 4 tasks (nest work, pulp foraging, carbohydrate foraging, and protein foraging) within a day for three days were compared: one day early in the study, one day mid-study, and one day late in the study. For the colony-level view, the same frequency distribution was calculated using the mean number of tasks per worker per day for the entire season.

**Figure 1.**
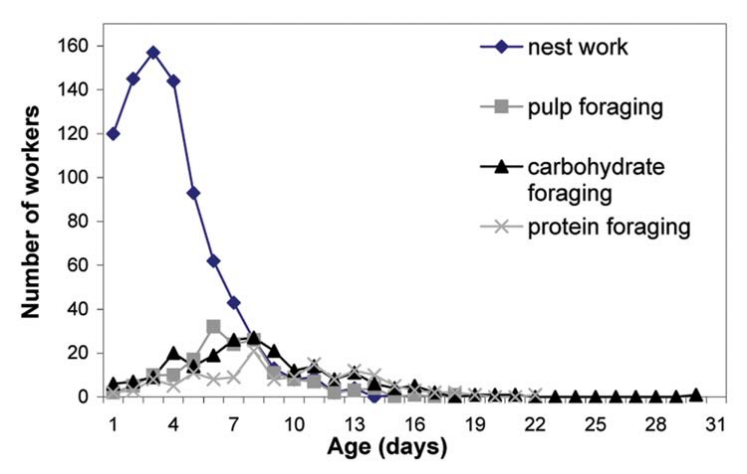
Frequency distribution of age of first performance of nest work, pulp foraging, carbohydrate foraging and protein foraging for workers of the 1998 colony. N = 215 known-age workers.

**Figure 2.**
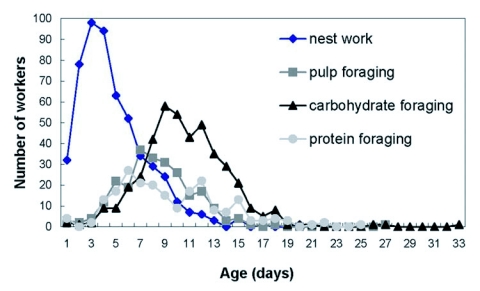
Frequency distribution of age of first performance of nest work, pulp foraging, carbohydrate foraging and protein foraging for workers of the 1999 colony. N = 573 known-age workers.

## Results

In early August of 1998 and 1999, the colonies were in the ergonomic stage of colony growth and rapidly produced workers. Males began to emerge in the last week of August, signifying that the colonies had switched to the reproductive phase. Workers added combs to nests, but the observation box constrained the nests' vertical growth. Thus the captive colonies did not grow to the sizes wild ones can.

**Table 1.**
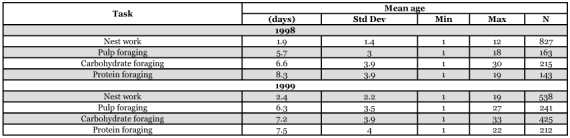
Average age at which workers first performed each kind of task.

From 28 July through 31 August of 1998 1,292 marked workers were observed performing nest-work, pulp, protein, or carbohydrate foraging. The ages of 215 of these workers were known. The unrelated workers introduced to the observation colony performed nest work and foraging tasks. The total number of marked workers observed between 27 July and 1 September 1999 was 1,924; 573 were of known-age. During August 1999 the mean ± SD value of the daily censuses of workers in the nest was 464 ± 114 workers. Size estimates for the last half of August 1999 are low however, because workers constructed carton chambers on the sides and top of the nest into which less active workers commonly retreated and were hidden from view.

### Estimates of worker lifespan for 1999 colony

For known-age workers of the 1999 colony seen on at least three different days and not seen after 24 August 1999 (one week before the study ended), the mean age ± SD (days) on the last day seen was 14.5 ± 5.9 days old (N = 428, range 3 30 days). The mean ± SD lifespan of workers seen at least once and not seen after 24 August 1999 was 12.1 ± 6.9 days (N = 554, range 1 – 30 days). Because it was possible that older workers may have become inactive on an obscured part of the nest and escaped observation between 24 August and 1 September, these averages are minimum estimates of life span.

**Table 2.**
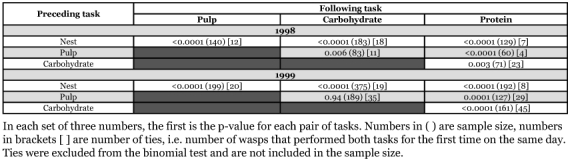
Results of binomial tests of the order of task performance based on age of first performance.

### Sequence and age of task performance

The “average” worker initially worked in the nest, and by the end of her first week as an adult began foraging for pulp, then carbohydrate, then protein. Some exceptional workers, however, foraged within a day or two after eclosion (Table 1). Workers typically ceased pulp foraging by the end of their third week. Carbohydrate foraging was the slowest to decline; some foragers continued to bring in carbohydrate until they were over 30 days old (Figures 1 and 2).

For both colonies, the differences in mean age at first performance of the different foraging tasks are relatively small (Table 1). Particularly in the 1999 colony, the differences in mean age between sequentially ranked task groups are all less than 1.5 days. Because of non-independence of data in Table 1, a direct statistical analysis was not possible. Binomial tests of equal probability of first occurrence for paired tasks found that workers in the 1998 colony foraged for pulp earlier than they foraged for carbohydrate (Table 2). There was no statistical difference in the starting age of foraging for pulp and carbohydrate in the 1999 colony. The sample sizes among tests differed because not all workers did all tasks. Of the 32 workers that met the criterion of foraging for each material at least three times, most (17/32) foraged for pulp before foraging for either carbohydrate or protein. Thirteen workers protein foraged before they carbohydrate foraged. Eleven carbohydrate foraged before they protein foraged. Eight workers foraged for both materials at approximately the same time. Three of those eight workers simultaneously foraged for pulp (Table 3).

**Table 3.**
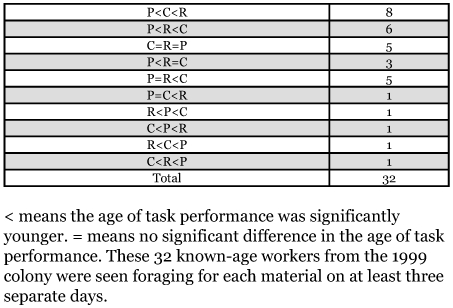
Numbers of workers that performed pulp (P), protein (R), and carbohydrate (C) foraging in different sequences over the course of their lives.

### Effect of time of season and body size on age of first foraging

To determine if time of season affected first-foraging age, the 1999 data were divided into four week-long blocks. Over this four-week period, the average age of first foraging increased by nearly six days (Table 4). The same analysis was done on the smaller, 1998 data set, and weekly variation was found in first-foraging age, but no general increase.

Multiple regressions of the body size measurements—weight, head size, thorax size, and the ratio of thorax size to weight—found no significant effect of any of these factors on the age of first foraging for either pulp or protein. However, workers with a larger thorax began carbohydrate foraging at a younger age than did smaller workers, although thorax size explained only a small fraction of the variability in age of first foraging (multiple regression: Adj. R^2^ = 0.15, N = 38, P = 0.009). The ratio of thorax size to weight had a negative, but small, effect only on age of first-carbohydrate foraging (multiple regression: Adj R^2^ = 0.11, N = 38, P = 0.02, Coeff. Var. = 41.4). Weight did not vary with age.

**Table 4.**
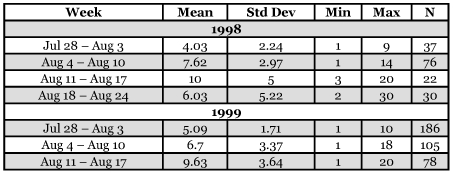
Mean ages of workers that carbohydrate foraged for the first time, by one-week intervals.

### Task specialization

Workers usually performed multiple tasks during their lives. Excluding nest work, 10% or fewer workers performed only one task (Table 5). There was no evidence that individuals abruptly switched from one task to the next. Some workers continued to work in the nest on days they foraged; some foraged for multiple materials on the same day.

**Figure 3.**
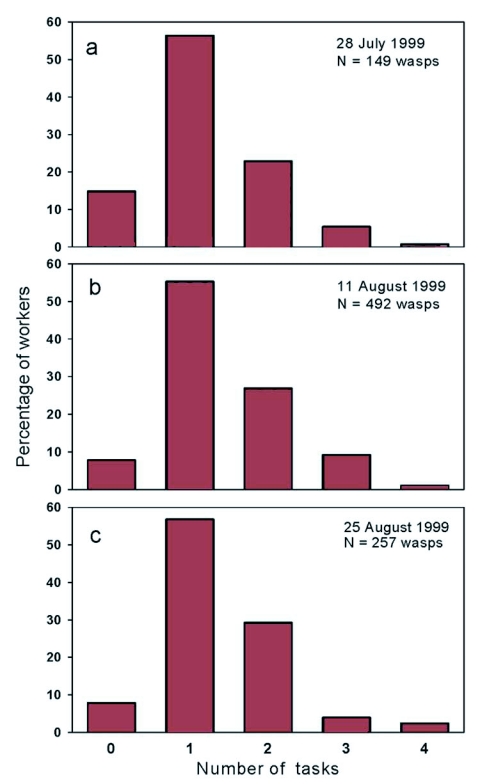
Percentage of workers that performed 0 1, 2, 3, or 4 tasks on a single day. a) 28 July, 1999, b) 11 August, 1999, c) 25 August, 1999. The daily proportions of workers performing different numbers of tasks are similar across days. Workers that worked multiple days contributed multiple observations.

**Figure 4.**
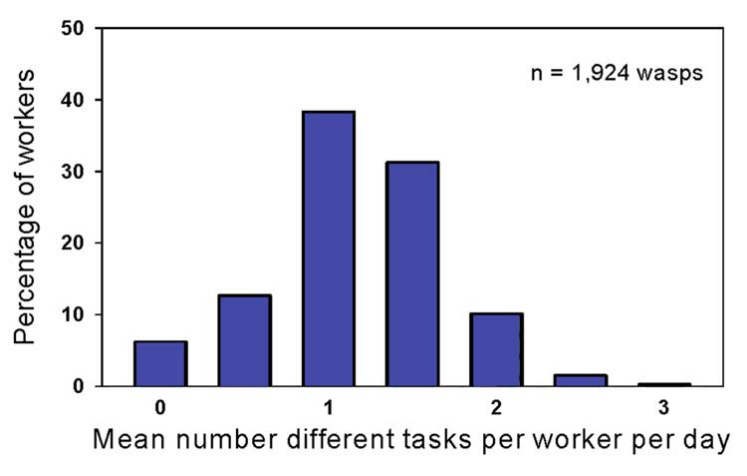
Average number of kinds of tasks per day per marked worker. 43% (827 wasps) averaged more than one task per day over the course of their lives.

The daily frequency distributions of the number of workers observed performing 0, 1, 2, 3, or 4 tasks appeared highly similar despite the colony's production of workers in July versus a mix of workers and reproductives at the end of August (Figure 3). The distribution of the lifetime average of number of tasks that workers performed per day peaked at one task and gradually declined as the number of tasks increased. Thirty-eight percent of the workers in the 1999 colony averaged approximately one type of task per day (although the kind of task changed), 43% averaged more than one task per day, and 19% averaged less than one task per day (Figure 4).

**Table 5.**
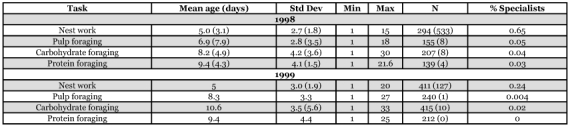
Mean task performance ages of workers that performed more than one task in their lives, with values for workers that performed only one kind of task given in parentheses.

**Figure 5.**
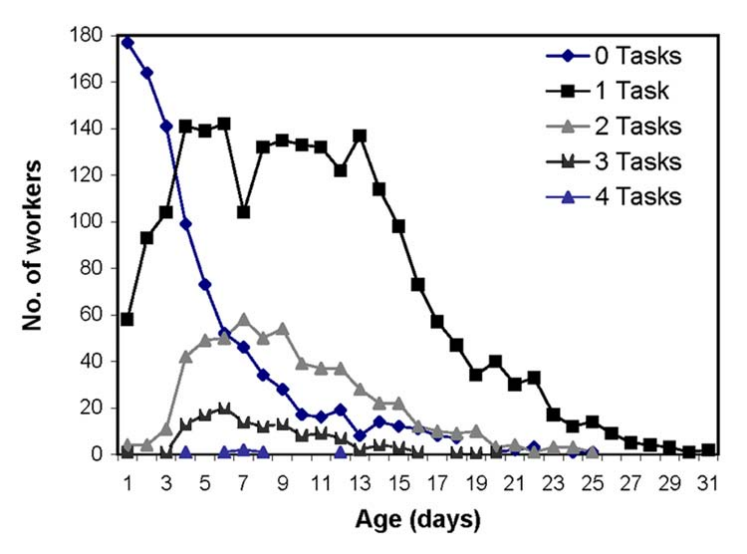
Frequency distribution showing the change in number of workers performing 0, 1, 2, 3, or 4 tasks over worker age.

The age distribution of numbers of tasks performed per day shows that the youngest workers were typically idle (Figure 5). Between ages four and twelve, the proportions of workers engaged in 1, 2, 3, and 4 different tasks were relatively constant and reflect the pattern shown in Figures 3 and 4. After day 14, due to worker mortality, the sample size was too small to discern a pattern. The distribution suggests that degree of task specialization was unrelated to age.

**Figure 6.**
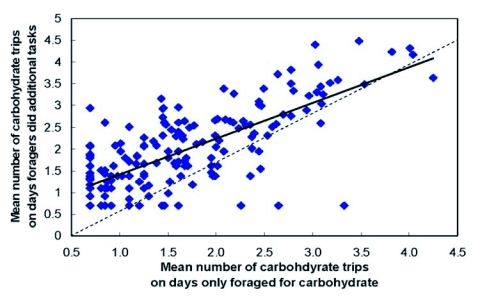
Mean number of carbohydrate trips that foragers made on days on which they performed multiple kinds of tasks as a function of the mean number of carbohydrate trips they made on days on which they only carbohydrate foraged. N = 191 foragers. Dashed line = null hypothesis; solid line = least squares regression.

Worker differences in degree of specialization do not necessarily mean that some workers are more productive than others. A less specialized forager that collects multiple materials may make the same number of foraging trips as her more specialized sister. For each carbohydrate forager that foraged solely for carbohydrate on some days and performed additional tasks on other days, her average number of carbohydrate trips made on days she performed additional tasks was regressed with the average number of carbohydrate-foraging trips made on days she was seen foraging only for carbohydrate. The null hypothesis was b_1_=1 and is shown in Figure 6 as the line through the origin. The fitted line in Figure 6 shows that most carbohydrate foragers made more trips on days when they did additional tasks (paired t-test: *t*_189_ = 3.26, P < 0.01, C I: %95). Thus, rather than making more trips on days they specialized on carbohydrate foraging, it appears that these workers had busy days and not-so-busy days. However, most workers made few carbohydrate trips regardless of whether they did other tasks that day.

## Discussion

The results suggest that *V. germanica* exhibits limited temporal polyethism. While approximately two-fifths of the *V. germanica* workers studied specialized on only one task per day, individuals varied in their age and sequence of task performance. Sample size and length of observation affected detection of a sequential progression of tasks. In the smaller, 1998 data set statistical support was found for the following task sequence: work in the nest, forage for pulp, forage for carbohydrate, and forage for protein. These results support the task progression reported in previous studies of *Vespula* species (Potter 1964; Spradbery 1973; Akre et al. 1976; Greene 1991). However, in 1999 the samples of marked workers and of observations per worker were much larger than in any prior studies, and no statistically significant evidence was found that workers perform pulp foraging prior to carbohydrate foraging. This suggests that sample size is relevant to considerations of temporal polyethism in *Vespula* species, although we had predicted that temporal polyethism would be more apparent in larger samples. Even though the ages of first performance differ statistically for some tasks, these differences are so small that they are of limited biological importance, particularly when compared to eusocial insect species with large colony sizes, such as honey bees and *P. occidentalis* wasps (Wilson 1971; Jeanne et al. 1988; Seeley 1995).

Examination of workers that foraged at least three times for all three materials during their lives demonstrates the relatively large variation in task performance sequences among foragers. Workers in this subset showed a tendency to forage for pulp before carbohydrate and protein, but were only slightly more likely to forage for carbohydrate before foraging for protein. The broader analyses shows that workers did not abruptly stop foraging for a material when they began to forage for a new material. Some foragers performed periodic nest work such as feeding larvae, fanning, or removing debris. Most adults began foraging for all materials approximately concurrently and performed tasks for less than two weeks. Therefore, it is unlikely that changes in the colony's need for materials greatly affected workers' sequence of tasks.

Worker mortality likely affected the number of tasks individuals were observed performing over the course of their lives. Given that nest work was most commonly the first task workers undertook, and the sample sizes of nest workers that performed only one task are larger than the sample sizes of foragers that performed only one task, it appears that many nest workers died before they could proceed to foraging tasks (Table 5). Premature death would explain why the mean age of first task performance was typically lower in workers that performed only one task compared to workers that performed multiple tasks during their lives.

The finding that the frequency distributions of the number of workers over the number of different tasks performed were highly similar across days suggests that the daily proportions of generalist to specialist workers stayed relatively stable throughout the study. This stability was maintained despite the colony's nearly three-fold increase in size, decreasing need for pulp, increasing need for carbohydrate, and its transition from rearing worker larvae to rearing reproductives. The source of this stability most likely lies in the specialist or generalist tendencies of individual workers. The distribution of lifetime average number of tasks per day per worker is highly similar to the colony-level distributions of specialists to generalists over separate days and across days. The relatively stable proportions of workers that do 1, 2, 3, or 4 tasks per day across a range of ages suggests that workers do not change their tendency to specialize as they age.

These results indicate that worker specialization in a *V. germanica* colony cannot be explained by a threshold - reinforcement mechanism. In individual - based simulation models, task specialization has been shown to emerge through the repeated interplay of two opposing factors operating on worker thresholds: 1) a worker's threshold to respond to task stimuli decreases after she performs the task and 2) her threshold increases as a function of time from her last performance of the task (Theraulaz et al. 1998). Such models are offered as explanations of elitism in social insects (Plowright and Plowright 1988; Gautrais 2002). Our study of the carbohydrate foragers in the 1999 colony found that successful retrieval of carbohydrate did not increase workers' rates of carbohydrate foraging (Hurd et al. 2003). Furthermore, the carbohydrate foragers that made the most trips, the elite foragers, were not more likely to be task specialists than those workers making few trips. In the present study, a positive relationship was found between carbohydrate-trip number and the number of different tasks performed by a carbohydrate forager, in direct contrast to predictions based on a self-reinforcement model.

Our finding of individual differences in workers' lifetime average number of tasks performed per day suggests a possible genetic effect on worker task specialization. Changes in gene expression have been shown to underlie temporal polyethism in honeybees (Page and Robinson 1991; Robinson et al. 1994; Page et al. 1995; Page and Erber 2002) and *Polybia* wasps (O'Donnell and Jeanne 1993; O'Donnell 1996). It is probable that *Vespula* workers have numerous genes in common with these species, but that these genes are expressed differently in *V. germanica*.

Specifics of life history likely play an important role in determining the timing and degree of worker specialization and the colony's ratio of specialists to generalists. In comparison to other flying hymenopteran species with large colony sizes, the temporal polyethism schedule followed by *V. germanica* wasps appears highly accelerated. Analysis of workers' ages of task performance found that most workers had begun foraging for all materials by the end of their first week and that some foragers also continued to perform nest work. These results are in contrast to *A. mellifera* bees and *P. occidentalis* wasps that typically switch abruptly and completely from nest tasks to foraging at the end of their third week, although the age of switching varies among individuals (Jeanne et al. 1988; Seeley 1995).

This acceleration may be related to worker life-span. We observed *V. germanica* workers for an average of 14.5 days before they disappeared and presumably died. Potter (1964) reported a similar life-span of *V. vulgaris* workers during the same stage of colony growth. Thus, these *Vespula* workers appear to live approximately half as long as *P. occidentalis* and *A. mellifera* workers (Jeanne et al. 1988; Seeley 1995). Perhaps *Vespula* workers do not live long enough for the colony to reap the efficiency benefits gained in repeated-task performance by specialized workers.

*Vespula* colonies in temperate zones have only a single summer to reproduce, another contrast to the longer-lived colonies of *P. occidentalis* and *A. mellifera*. To rear large numbers of reproductives in a single season, *Vespula* colonies must rapidly produce a labor force of workers. Because initial colony-sizes of *Vespula* species are small since they are started by a single founding queen, a strategy of labor allocation that utilizes a relatively large proportion of generalist workers may be the most productive for young colonies. Larger colonies are better able to defend themselves against predators, find food, and thermoregulate than small colonies (Wilson 1971). A growing *V. germanica* colony will achieve these benefits faster if it allocates incoming resources to the production of additional short-lived workers, rather than to the maintenance of fewer long-lived workers.
